# Young adults’ lived experiences of parental cancer: a qualitative evidence synthesis

**DOI:** 10.1016/j.ijnsa.2026.100531

**Published:** 2026-04-06

**Authors:** Rikke Guldager, Trine Andersen, Helle Sørensen von Essen, Mette Gothardt Lundh, Karin Bundgaard, Tina Wang Vedelø

**Affiliations:** a Department of Neurosurgery, Copenhagen University Hospital – Rigshospitalet, Copenhagen, Denmark; b Department of Neurosurgery, Odense University Hospital, Odense, Denmark; c Department of Clinical Research, University of Southern Denmark, Denmark; d Department of Intensive Care, Copenhagen University Hospital- Rigshospitalet, Copenhagen, Denmark; e Clinical Nursing Research Unit, Aalborg University Hospital, Aalborg, Denmark; f Department of Clinical Medicine, Aalborg University, Aalborg, Denmark; g Research Unit of Nursing and Health Care, Aarhus University, Aarhus, Denmark; h Department of Neurosurgery, Aarhus University Hospital, Denmark

**Keywords:** Cancer, Caregiving, Experience, Parental, Qualitative, Young adults

## Abstract

**Background:**

Young adults aged 18 to 35 years with a parent affected by cancer often face emotional, financial, and social burdens. Parental illness disrupts this transitional life stage, which is commonly characterised by education, career-building, and relationships, by adding caregiving responsibilities that may strain identity, well-being, and social connections. Despite their psychosocial vulnerability, young adult caregivers remain underrepresented in research, and little is known about how they experience and navigate these challenges.

**Objective:**

To address this gap, this systematic review identifies and synthesises qualitative research on individuals who experience parental cancer during young adulthood or emerging adulthood.

**Information sources:**

The literature search was conducted in MEDLINE, Embase, CINAHL, PsycINFO, Cochrane Library, Google Scholar, Web of Science, and Scopus, from inception to May 2024, with an updated search in September 2025. In addition, the reference lists of included studies were screened to identify further eligible records.

**Methods:**

We conducted a qualitative evidence synthesis following established methodological and reporting guidelines (PRISMA-P and ENTREQ). The review included qualitative studies and data were synthesised using thematic synthesis as described by Thomas and Harden.

**Results:**

The included studies, conducted in Asia, Europe, North America, and Oceania, used predominantly qualitative or mixed methods and were published in English between 2008 and 2024. The results indicate that young adults live everyday lives that unfold in parallel with the medical trajectory of their parent, a process characterised by shifting social roles, disrupted relationships, and an evolving sense of identity. These experiences coalesce into three overarching themes: (1) *emotional burden*, encompassing the impact on young adults’ emotions and identity; (2) *balancing family dynamics*, involving the navigation of conflicts, fostering of closeness, and management of responsibilities; and (3) *effective management strategies*, highlighting the role of support, access to information, and active involvement in caregiving.

**Conclusion:**

Parental cancer profoundly affects young adults, influencing their emotional well-being, personal identity, and family responsibilities. While these challenges can be overwhelming, they may also foster personal growth and resilience. Difficulties in seeking support underscore the need for tailored interventions to help young adults maintain well-being.

**Registration:**

The review protocol was registered with PROSPERO, the International Prospective Register of Systematic Reviews 23 December 2023. Registration number CRD42023492854

**What is already known**

• Young adults with a parent diagnosed with cancer face emotional, social, and developmental challenges.

• Parental cancer disrupts key life stages, such as education, career, and relationships, creating role strain and caregiver burden.

• There is growing recognition of the need for tailored support, but specific evidence for this group is still limited.

What this paper adds

• This synthesis identifies emotional burden, shifting identities, and caregiving roles.

• It delineates how parental cancer disrupts family dynamics and life stages.

• Strategies for managing caregiving and support among young adults are revealed in the synthesis.

Alt-text: Unlabelled box dummy alt text

## 1 Introduction

Young adult caregivers providing care for a parent with cancer face significant emotional, financial, and social burdens, as the illness affects not only the patient, but the entire family (Alfaro-Díaz et al., 2022; Chen et al., 2015; Soerjomataram and Bray, 2021). With 19.3 million new cancer cases worldwide in 2020, a substantial number of young adults are thrust each year into caregiving roles and forced to navigate challenges that can shape their well-being and future opportunities (4–7).

Young adults, defined in this review as individuals aged 18–35 years, in accordance with Arnett’s theory of Emerging Adulthood (Arnett, 2000), are in a life stage characterized by significant physical, emotional, and psychological transitions (Bystrup et al., 2023). This developmental period represents a shift from childhood dependency to adult independence (Society for Adolescent Health and Medicine, 2017; Journals Editors, 2011), and is commonly characterised by engagement in education, early career development, and the formation of intimate relationships (Pope et al., 2022). Young adulthood is further characterized by cognitive individualism and a gradual transition toward stability, including assuming responsibility for oneself, developing independent decision-making, forming and maintaining intimate relationships, establishing families, consolidating career paths, and achieving financial independence (Arnett, 2000).

Parental cancer disrupts this period, creating role strain as young adults juggle developmental tasks with caregiving responsibilities, leading to emotional and physical stress (Pope et al., 2022; Compas et al., 1994; Compas et al., 1996). Even young adults not formally recognised as caregivers often take on new caregiving duties (Waters et al., 2021). Parental cancer refers to a young adult having a mother or father diagnosed with cancer, regardless of type or stage. This focus highlights the unique developmental and relational role parents play in young adults’ lives, as illness can alter family roles, dependencies, and responsibilities. The term “child” includes biological, adoptive, foster, and stepchildren.

These impacts constitute the lived experience of young adult caregivers, reflecting their perspectives on being affected by parental cancer. Lived experience encompasses their emotional, social, and practical challenges, including their support preferences, unmet needs and caregiving experiences (Ghofrani et al., 2019). Previous studies indicate that parental cancer impacts young adults through caregiver burden, depression, and changes in social relationships (Chen et al., 2018; Pope et al., 2022; Chevrier et al., 2022; McDonald et al., 2016; Saragosa et al., 2022). Those who take on caregiving responsibilities often have less time for family and friends, forgo social activities, and experience peer disconnection. (Pope et al., 2022; Rose and Cohen, 2010). Caregiving roles range from part- to full-time, depending on family structure and the parent’s functional status (Chevrier et al., 2022). Although young adult caregivers are more likely than older caregivers to combine work and caregiving, they are still less likely to be employed than peers without caregiving duties (Warner et al., 2021). Despite recognition of their psychosocial vulnerability, young adult caregivers remain an understudied group, with limited research exploring these lived experiences in depth. To address this gap, this systematic review identifies and synthesises qualitative research exploring the lived experiences of young adults affected by parental cancer during young adulthood or emerging adulthood.

## 2 Aim

The aim of this review is to systematically identify and synthesise qualitative studies exploring the experiences of individuals whose parents have cancer during young or emerging adulthood. It addresses the following questions:

1) What is the lived experience of being a young adult caregiver for a parent with cancer?
2) How do young adult caregivers describe their everyday lives, including changes in roles, jobs, education, and relationships with others?

## 3 Design and methods

This systematic review focuses on qualitative research studies to achieve an in-depth and participant-oriented understanding of young adults’ lived experiences with parental cancer. Therefore, we conducted a qualitative evidence synthesis (QES), a method used to deepen understanding of a specific phenomenon of interest (Flemming and Noyes, 2021).

### 3.1 Protocol registration and reporting guidelines

The review protocol was registered with PROSPERO, the International Prospective Register of Systematic Reviews, under the registration number CRD42023492854. This systematic review is reported by the Preferred Reporting Items for Systematic Reviews and Meta-analysis for Protocols (PRISMA-P) (26). It adheres to the methods described by Flemming and Noyes (Noyes et al., 2023) and is reported following the Enhancing Transparency in Reporting the Synthesis of Qualitative Research (ENTREQ) guidelines (Tong et al., 2012).

### 3.2 Search methods

An extensive literature search was undertaken across MEDLINE (Ovid), Embase (Ovid), CINAHL (EBSCO), PsycINFO, and Google Scholar in May 2024 and updated on September 4, 2025. We also searched the citation databases Web of Science and Scopus for relevant records citing the included studies. Finally, we searched MEDLINE for publications by first and last author and screened the reference lists of included studies for additional relevant records.

#### 3.2.1 Search strategy

The search strategy was developed by the authors in collaboration with an information specialist. The initial search strategy was developed in MEDLINE (Ovid) using a combination of index terms and controlled vocabulary. Subsequently it was adapted and tailored to the remaining databases (Appendix I).

### 3.3 Inclusion and exclusion criteria

#### 3.3.1 Inclusion criteria

We included studies exploring the lived experience of young adults (18–35 years) with a parent diagnosed with cancer (Arnett, 2000). In cases where a study’s age range exceeded the predefined interval, the study was included if the majority (≥50%) of participants were within the 18 to 35 age range or if qualitative data specific to this age group could be extracted separately. Due to inconsistent age reporting in the studies, this pragmatic eligibility approach was applied.

We included primary qualitative studies employing qualitative methods for data collection and analysis. Mixed methods studies and multi-methods studies were included if qualitative data could be extracted separately. For pragmatic reasons, we included only studies published in English and Scandinavian languages. No restrictions were applied regarding the study setting or year of publication.

#### 3.3.2 Exclusion criteria

Studies exploring experiences of parental cancer occurring during childhood (i.e., before the age of 18 years) were excluded. Literature reviews, single descriptions, conference abstracts, and reports were also excluded.

### 3.4 Search outcome and study selection

#### 3.4.1 Study inclusion

All identified records (*n* = 36,662) were imported into Covidence (Veritas Health Innovation, Melbourne, Australia), and duplicates were removed, leaving *n* = 23,772. Four authors independently assessed the records for inclusion in two steps: First, titles and abstracts were screened for obvious exclusion, and second, the full text was assessed for final inclusion. At each stage of the selection process, any disagreements among the reviewers were resolved through discussion. Explanations for study exclusions at each stage were documented in the PRISMA flow diagram (Table 1).

**Table 1:** Young adults’ lived experiences of parental cancer: a systematic review of qualitative studies.

Following the reviewer’s comments, all included studies were re-examined to verify age eligibility and clarity of age reporting.

#### 3.4.2 Data extraction

In pairs of two, the authors independently extracted data using a data extraction form developed in Microsoft Excel 2016 (Appendix II). The extractions followed a two-stage process: (1) extraction of “contextual” details (e.g., general information and population) and (2) extraction of “findings” (Flemming and Noyes, 2021). The data extraction form was initially tested on three studies, and small changes were made. Any discrepancies in extractions were discussed by the author pair; in case of persistent disagreement, a third author was consulted.

### 3.5 Quality appraisal

Eligible studies were critically assessed for methodological quality by two authors individually using the Critical Appraisal Skills Programme (CASP) checklist for qualitative research (Critical Appraisal Skills Programme, 2023). Studies were included if they received a “yes” response to at least 50% of the CASP questions (Batten and Brackett, 2021). Any disagreements were resolved through discussion.

### 3.6 Analysis

#### 3.6.1 Data synthesis

For the QES, we employed a thematic synthesis, as described by Thomas and Harden (Thomas and Harden, 2008) and recommended by The Cochrane Qualitative Review Methods Group, to inductively analyse the data extracted from each study (Noyes et al., 2023). This process comprised a three-stage method, including line-by-line coding of the extracted data, development and categorisation of descriptive themes, and generation of analytic themes that went ‘beyond’ the results reported in the original studies (Flemming and Noyes, 2021). Four authors (RG, TA, HSE, and TWV) independently coded the results of all included studies, and the independent coding was cross-checked to ensure the comprehensiveness of the assigned codes. The codes were then grouped into descriptive themes. Next, the first author and last author (RG and TWV) created analytical themes, which were reviewed and discussed with authors TA, KB, and HSE until consensus on the final themes was reached. Through discussions, the GRADE-CERQual approach was employed to assess confidence in the findings of the QES (Lewin et al., 2018).

## 4 Results

### 4.1 Quality assessment

Ultimately, 12 studies were determined to have high methodological quality based on their CASP scores (Table 2) and were included in the review. The included studies clearly stated their research aims and demonstrated consistency across methodology, research objectives, data collection, representation, and analysis. For five of the included studies, potential bias concerning the relationship between the researcher and the participants was considered and discussed.

**Table 2:** Summary of the methodological quality and dependability of eligible studies (*n* = 12).

Criteria	Almarza (2008)	Bergersen et al. (2022)	Fang et al. (2022)	Fletcher et al. (2019)	Fujimoto & Kanda (2023)	Goldblatt et al. (2019)	Karlsson et al. (2013)	Kastrinos et al. (2023)	McPhail et al. (2017)	Puterman & Cadell (2008)	Tong et al. (2024)	Tulpin et al. (2024)
1. Was there a clear statement of the aims of the research?	Yes	Yes	Yes	Yes	Yes	Yes	Yes	Yes	Yes	Yes	Yes	Yes
2. Is a qualitative methodology appropriate?	Yes	Yes	Yes	Yes	Yes	Yes	Yes	Yes	Yes	Yes	Yes	Yes
3. Was the research design appropriate to address the aims of the research?	Yes	Yes	Yes	Yes	Yes	Yes	Yes	Yes	Yes	Yes	Yes	Yes
4. Was the recruitment strategy appropriate to the aims of the research?	Yes	Yes	Yes	Yes	Yes	Yes	Yes	Yes	Yes	Yes	Yes	No
5. Was the data collected in a way that addressed the research issue?	Yes	Yes	Yes	Yes	Yes	Yes	Yes	Yes	Yes	Yes	Yes	Yes
6. Has the relationship between researcher and participants been adequately considered?	Yes	Yes	No	No	No	No	Yes	No	Yes	Yes	No	No
7. Have ethical issues been taken into consideration?	Yes	Yes	Yes	Yes	Yes	Yes	Yes	Yes	No	Yes	Yes	Yes
8. Was the data analysis sufficiently rigorous?	Yes	Yes	Yes	Yes	Yes	Yes	Yes	Yes	Yes	Yes	Yes	Yes
9. Is there a clear statement of findings?	Yes	Yes	Yes	Yes	Yes	Yes	Yes	Yes	Yes	Yes	Yes	Yes

Note: Items 1 to 10 are the criteria in the JBI critical appraisal checklist.

### 4.2 Study characteristics

In total, our database search identified 23,772 unique records. By reading titles and abstracts, we excluded 23,619 records, leaving 153 records for which we assessed the full text. Of these records, 139 were excluded and 12 were retained for further assessment (Table 1). The final 12 studies were conducted in Asia (25%), Europe (25%), North America (42%), and Oceania (8%). All were qualitative (92%) or part of mixed methods research (8%) and were published in English between 2008 and 2024, with most published in 2022 (33%). Data were primarily collected through semi-structured (83%), autoethnographic (8%), and narrative interviews (8%). The analytical approaches employed comprised thematic analysis (42%), content analysis (25%), grounded theory (25%), and phenomenological analysis (8%). The participants were recruited through healthcare providers and institutions (33%), physical and online media and advertisement (33%), or both (33%). Recruitment methods varied, including purposeful sampling (50%), theoretical sampling (8%), convenience sampling (8%), and self-selection sampling (33%) (Table 3).

**Table 3:** Summary of the methodological quality and dependability of eligible studies (*n* = 12).

Criteria	Almarza (2008)	Bergersen et al. (2022)	Fang et al. (2022)	Fletcher et al. (2019)	Fujimoto & Kanda (2023)	Goldblatt et al. (2019)	Karlsson et al. (2013)	Kastrinos et al. (2023)	McPhail et al. (2017)	Puterman & Cadell (2008)	Tong et al. (2024)	Tulpin et al. (2024)
1. Was there a clear statement of the aims of the research?	Yes	Yes	Yes	Yes	Yes	Yes	Yes	Yes	Yes	Yes	Yes	Yes
2. Is a qualitative methodology appropriate?	Yes	Yes	Yes	Yes	Yes	Yes	Yes	Yes	Yes	Yes	Yes	Yes
3. Was the research design appropriate to address the aims of the research?	Yes	Yes	Yes	Yes	Yes	Yes	Yes	Yes	Yes	Yes	Yes	Yes
4. Was the recruitment strategy appropriate to the aims of the research?	Yes	Yes	Yes	Yes	Yes	Yes	Yes	Yes	Yes	Yes	Yes	No
5. Was the data collected in a way that addressed the research issue?	Yes	Yes	Yes	Yes	Yes	Yes	Yes	Yes	Yes	Yes	Yes	Yes
6. Has the relationship between researcher and participants been adequately considered?	Yes	Yes	No	No	No	No	Yes	No	Yes	Yes	No	No
7. Have ethical issues been taken into consideration?	Yes	Yes	Yes	Yes	Yes	Yes	Yes	Yes	No	Yes	Yes	Yes
8. Was the data analysis sufficiently rigorous?	Yes	Yes	Yes	Yes	Yes	Yes	Yes	Yes	Yes	Yes	Yes	Yes
9. Is there a clear statement of findings?	Yes	Yes	Yes	Yes	Yes	Yes	Yes	Yes	Yes	Yes	Yes	Yes

Note: Items 1 to 10 are the criteria in the JBI critical appraisal checklist.

The majority of participants were female (77%), with gender not reported in one study. The average age at the time of parental diagnosis was 21.9 (15.3–27), though this was not reported in nine studies. At the time of the interview, the average age was 23.6 (17.1–35), however, this was not reported in three studies. The young adults were mainly students and employed (33%) or students (17%), with occupational details missing in six studies. Housing situations included living at home with their parent (33%) or a mix of living at home and away (42%), though this was unreported in three studies. Four participants were primary caregivers, while in five studies, another relative (often a partner) held this role. This was not reported in three studies. Other family members mentioned included spouses/partners, siblings, other relatives, and others.

In 68% of the studies, the parents were female, though only one study reported their age at diagnosis, and none reported age at the time of the interview. Parental diagnoses included various cancers; pancreatic cancer, lung cancer, brain cancer, bowel cancer, ovarian cancer, cervical cancer, bone marrow cancer, neuroendocrine cancer, kidney cancer, testicular cancer, and non-Hodgkin’s lymphoma, breast cancer, unknown cancer, and unreported diagnoses (Table 4).

**Table 4:** Key participant characteristics (study-level reporting).

Young adults’ characteristics		Studies and participants included (%)^a^
Total participants		160
Median (range) per study		13 (3–33)
Sex (female) %, mean (range)^b^		77 (50–100)
Age at parents’ diagnosis, mean (range)^c^ Age at time of interview, mean (range)^d^ Occupation Housing situation Primary caregiver	Student, employed Student Employed Not reported Living at home with parent Living at home with parent, Living away from parents Not reported Yes No Not reported	21.9 (15.3–27) 23.6 (17.1–35) 4 (33) 2 (17) 0 (0) 6 (50) 4 (33) 5 (42) 0 (0) 3 (25) 4 (33) 5 (42) 3 (25)
Parents’ characteristics		
		
Total number, median (range) Sex (female) %, mean (range)^e^		NA 68 (0–100)
		
Age at diagnosis, mean (range)^f^ Age at time of interview, mean (range)^g^		NR - (-)
Parent’s diagnosis Other relatives than the young adult	A variety of cancer diagnosis’, being pancreatic, lung, brain, bowel, ovarian, cervical, bone marrow, neuroendocrine, kidney, testicular, and Non-Hodgkin’s lymphoma Breast cancer Cancer (unknown type) Not reported Spouse/partner Yes No Not reported Other children Yes No Not reported Other family Yes No Not reported Others Yes No Not reported	7 (58) 2 (17) 3(25) 0 (0) 8 (67) 0 (0) 4 (33) 6 (50) 0 (0) 6 (50) 5 (42) 0 (0) 7 (58) 4 (33) 0 (0) 8 (67)

^a^ Percentages do not always add up to 100 due to rounding.

^b^ Not reported in one study.

^c^ Not reported or not applicable in nine studies.

^d^ Not reported or not applicable in three studies.

^e^ Not reported or not applicate in six studies.

^f^ Only one study reported the parents’ age at the time of diagnosis; range not reported.

^g^ None of the studies reported the parents age at the time for the interview.

### 4.3 Synthesis of results

We identified nine descriptive themes characterising young adults’ experiences of living with parental cancer, and we consolidated these into three analytic themes. These synthesised findings reflect: (1) emotional burden: the impact of having a parent with cancer on young adult caregivers’ emotions and identity; (2) balancing family dynamics: navigating conflicts, fostering closeness, and managing responsibilities; and (3) effective management strategies: the role of support, information, and active involvement.

#### 4.3.1 Theme 1: emotional burden: the impact of having a parent with cancer on young adult caregivers’ emotions and identity

*The emotional burden of having a parent with cancer*

Young adult caregivers experienced profound suffering when their parent was diagnosed with cancer, and experiences of grief, fear of loss, anxiety, and distress were common (ALMARZA and SMITH, 2010; Fujimoto and Kanda, 2023; Fang et al., 2022; Karlsson et al., 2013; Puterman and Cadell, 2008; Fletcher et al., 2019; Tulpin et al., 2023; McPhail et al., 2017; Tong et al., 2024).

*The young adults experienced grief from the moment the parent was diagnosed with cancer. A sense of hopelessness arose even before they knew the prognosis.* (*KARLSSON et al., 2013*)*.*

Others emphasised a disrupted sense of faith and a deep feeling of unfairness:

*My mom is such a good person [crying] and it’s not fair that she doesn’t get to live as long as other people who aren’t as nice or generous or strong as she is. (**TONG et al., 2024**).*

Some young adult caregivers reported becoming more health conscious after their parent’s cancer diagnosis, but others experienced concerns about their own health, particuarly concerns about having cancer themselves (McPhail et al., 2017). These concerns were most commonly related to types of familiar types of cancer, such as breast cancer (Fujimoto and Kanda, 2023; Fang et al., 2022). Persistent and evolving thoughts about cancer sometimes dominated their mental landscape, compelling them to navigate the tension between maintaining hope and confronting the fear of losing a parent (ALMARZA and SMITH, 2010; Kastrinos et al., 2023).

These concerns added to the young adult caregivers’ emotional burden and uncertainty about the future. As a result, many experienced emotional isolation, choosing to bottle up their struggles to avoid burdening their already ill parent (ALMARZA and SMITH, 2010; Fang et al., 2022; Fujimoto and Kanda, 2023; Fletcher et al., 2019).

*The daughters shielded themselves from anxiety and impatience by distraction and persuasion. Some of them were unable to express their feelings and closed themselves off: “No, I have not consulted with anyone other than my family. Maybe, I did not even talk about it with my family. Well, I do not want to think about it.” (**FUJIMOTO and KANDA, 2023**).*

Additionally, young adult caregivers felt that their parents tried to protect them by withholdig information about the severity of the illness. This contributed to their sense of isolation and distress (Bergersen et al., 2022).

*Navigating care responsibilities, guilt, and boundaries*

When young adults took on caregiving responsibilities for a parent with cancer, they underwent significant emotional changes, as highlighted in all reviewed studies (ALMARZA and SMITH, 2010; Bergersen et al., 2022; Fang et al., 2022; Fujimoto and Kanda, 2023; Karlsson et al., 2013; Puterman and Cadell, 2008; Fletcher et al., 2019; McPhail et al., 2017; Tulpin et al., 2023; Tong et al., 2024; Goldblatt et al., 2019). This shift in roles disrupted their daily lives, profoundly affecting their emotional well-being and mental health (ALMARZA and SMITH, 2010; Bergersen et al., 2022; Fang et al., 2022; Fujimoto and Kanda, 2023; Goldblatt et al., 2019; Karlsson et al., 2013; Puterman and Cadell, 2008; Fletcher et al., 2019; Kastrinos et al., 2023; McPhail et al., 2017). Young adult caregivers often found themselves struggling to balance the demands of caregiving with their own needs and boundaries (Puterman and Cadell, 2008). This balance was frequently disrupted by feelings of guilt, which stemmed from a perception of not doing or being there enough for their parent (Bergersen et al., 2022; Fletcher et al., 2019; Tong et al., 2024).

*I wasn’t a support system for [diagnosed mom], and that’s what I felt the worst stuff about—that I couldn’t be there for her when she’s always been there for me when I’ve been in trouble or anything or sick. And I just felt really bad. (**KASTRINOS et al., 2023**).*

In some cultures, young adult caregivers faced expectations that framed caregiving as an obligation, with children expected to care for ill parents regardless of personal desires or emotional readiness (ALMARZA and SMITH, 2010). Some reported difficulty identifying with the label of ‘caregiver’, despite resonating with the experience itself. This difficulty stemmed from perceiving their role as a duty or mandate within a cultural or religious context (Tong et al., 2024). As one participant explained:

*In our culture, the girl does all the household work. It’s not really expected of a man at all. It is sometimes very overwhelming and draining (Tong, 2024, P. 1062).*

This sense of duty influenced how young adults perceived their role and navigated their caregiving responsibilities. One young adult caregiver shared how she felt compelled to act as the ‘gatekeeper’ in her family, trying to maintain peace and prevent situations from escalating (Goldblatt et al., 2019). Even in her mother’s final days, she was caught between opposing family desires, navigating her mother’s wish for a secular funeral and her father and brother’s preference for a religious ceremony. Fulfilling these conflicting expectations left her feeling trapped and isolated (Goldblatt et al., 2019).

*Impact on identity and life priorities*

Beyond the immediate struggle of caregiving, young adult caregivers experienced profound and lasting changes in their identity and life priorities (McPhail et al., 2017; Tulpin et al., 2023; Tong et al., 2024). Many felt compelled to *‘grow up’* prematurely, assuming responsibilities that set them apart from their peers and fundamentally altered their transition into adulthood (Bergersen et al., 2022; Karlsson et al., 2013; Tong et al., 2024).

*I think when you hear that someone in your family whom you love has cancer, makes you change forever. Before you are happy and you don’t think that anything wrong can happen, anything bad, nothing really serious. Little things are bad here and there, but not serious. And then something like this happens. And you see a new part of something that you never saw before. Something very serious (*Almarza, 2008*).*

In addition, the experience of having a parent with cancer shifted young adults’ perspectives on life, death, and relationships and transformed their worldview and sense of self (Tong et al., 2024). The shift in self-perception also changed how they approached life and death (Goldblatt et al., 2019; Karlsson et al., 2013; Fletcher et al., 2019). As one young adult caregiver experienced, taking on the role of primary caregiver strengthened her ability to confront death and reshaped her perception of pain and loss:

*After the event, she was able to face death and considered it as an integrative part of life. She was capable of containing negative emotions, associated with end-of-life issues, and felt competent enough to support other people enduring pain and terminal illness (**GOLDBLATT et al., 2019**).*

#### 4.3.2 Theme 2: balancing family dynamics: navigating conflicts, fostering closeness, and managing responsibilities

*Family dynamic in transition*

Parental cancer significantly altered family dynamics, and young adult caregivers frequently experienced a reversal of traditional roles (Bergersen et al., 2022; Fang et al., 2022; Fujimoto and Kanda, 2023; Tulpin et al., 2023; McPhail et al., 2017; Tong et al., 2024). Many described a sudden need to assume responsibilities previously managed by their parents, in addition to providing direct care. These responsibilities often extended beyond household tasks to include financial obligations, such as seeking employment, contributing to family bills, and budgeting, particularly when the ill parent was the primary provider (Tong et al., 2024).

*You’re suddenly thrust into a position of being their parent earlier than you should have to and… worrying about having to figure out things, like financial things, or the “What do I do when?” … suddenly I feel like I’m going to have to take over control of the family somehow. Like, it’s almost having to grow up too fast even though we are grown up. But suddenly a lot of responsibilities are on you that shouldn’t be yours yet (**PUTERMAN and CADELL, 2008**).*

In addition, the altered awareness of their parent’s mortality and the changed family dynamics disrupted young adult caregivers’ sense of security (ALMARZA and SMITH, 2010). Some experienced their caregiving role as a personal calling or necessity, feeling that they had no choice other than to take on the role (Bergersen et al., 2022).

*My mother kept saying: “I will get through this stage thanks to you… when I die, you will get your life back.” I gave up a great deal of my life, but for my mother’s sake… at this stage, it was obviously my responsibility to take care of her… I did not choose this role—I was chosen and was led into that situation. I never asked myself: “What am I doing?”… The whole burden seemed to be on my shoulders… I was a pleaser; a “by-the-book” daughter; my decision to take on the role was convenient for both my parents. I perceived my role as a mission (Goldblatt, 2019, P. 536).*

For others, the role evoked feelings of resistance or resentment (Goldblatt et al., 2019).

*How much can a young woman take? Wiping his ass, opening his stoma… I never learned to perform these procedures. Why me?! Even the private nurse refused to touch that. I never chose to be a nurse… it is so disgusting to see a person vomiting. But, what can you do, can you leave him lying in his own shit? [Despite this,] I took on the emotional aspect of caring (Goldblatt, 2019, P. 537).*

*The challenge of balancing caregiving and personal aspirations*

Young adult caregivers often navigated multiple and, at times, conflicting responsibilities, necessitating the development of effective time management and prioritisation strategies (Fujimoto and Kanda, 2023; Fletcher et al., 2019). This challenge was further intensified by external pressures from family, friends, and society, which could inadvertently heighten their sense of duty or exposure to judgment (McPhail et al., 2017; Fujimoto and Kanda, 2023; Fang et al., 2022; Kastrinos et al., 2023). A strong desire to maintain a sense of normalcy frequently led to difficulties in reconciling caregiving duties with personal interests, social activities, and academic or career ambitions (McPhail et al., 2017; Fletcher et al., 2019).

Young adult caregivers often faced tension between caregiving responsibilities and the pursuit of a ‘normal’ life, leading to internal conflict and heightened stress. As a result, young adult caregivers reported coping through professional counseling, communication with significant others, avoidance by focusing on normality, and acceptance (Fletcher et al., 2019; McPhail et al., 2017). One young adult illustrated this strategy of avoidance and normalisation as follows:

*My tendency is to kind of deal with things by removing or distancing myself… I think it’s important for all of us to do our own things and move on with our lives and not dwell on it so much… For me, it’s just as nice to go home and hang out with my parents and treat it like it’s any other day.’ (**MCPHAIL et al., 2017**).*

Many young adult caregivers reported feeling pressure not only to care for their ill parent, but also to support healthy parents and family, further straining their ability to maintain their well-being:

*Now I am spending more time with them. For example, before I wanted to move out of the house, whether for school, but just outside the house, because of the independence. Now I’d rather just stay home and be with my parents [long silence]. I guess it just made me revolve around my family (Almarza, 2010, P. 139).*

The challenge of balancing caregiving with personal aspirations was evident in many young adult caregivers’ decisions regarding education and career. Some struggled with motivation and chose to discontinue their studies, while others remained determined to pursue their education and career goals while integrating caregiving into their daily lives:

*I decided that I would not stop my studies as well as work. Since we did not know how long it would last, I integrated the duty of caregiving for my father and eventually I even completed my studies although the last month was difficult (Goldblatt, 2019, P. 536).*

*Closeness and conflicts*

Being a young adult caregiver entailed both enhanced closeness and conflict within the family (ALMARZA and SMITH, 2010; Goldblatt et al., 2019; Tulpin et al., 2023; McPhail et al., 2017; Tong et al., 2024). Many young adult caregivers reported that their relationships with family members, particularly the ill parent deepened as they prioritised time together, fostering a heightened sense of closeness and appreciation for shared time (Fletcher et al., 2019). This priorisation of family time often contrasted with the lifestyles of the friends. As one young adult caregiver reflected:

*Other people [at university] just want to go away or go out and party. … I’m having dinner with my parents on Saturday and that’s okay. For especially casual acquaintances, they want you to go out with them. It can be hard to explain why it’s just so important tha you actually go home and see your family (McPhail, 2027 P209).*

The sense of closeness was linked to a shift in family dynamics and a heightened appreciation for relationships and shared time with family members. However, the intense emotional burden, coupled with differing expectations and needs, could lead to misunderstandings, tension, and conflicts (Fang et al., 2022; Goldblatt et al., 2019; Tong et al., 2024).

*My father wanted to stay only in the living room, and everyone had to accept that… I never invited friends to this house… He would always ask: “Where are you going?! What are you doing?” Home became a prison. I felt trapped, with no freedom… because a sick man becomes an egoist… there were moments when I just wanted to kill him. It might sound crazy, but I wanted to do that (Goldblatt, 2019, P. 537).*

#### 4.3.3 Theme 3: effective management strategies: the role of support, information, and active involvement

*The critical role of receiving support from family, friends, and healthcare professionals*

The young adult caregivers emphasised support from friends, family, healthcare professionals, and peers as essential to fostering a sense of safety and managing the emotional strain associated with caregiving (ALMARZA and SMITH, 2010; Bergersen et al., 2022; Karlsson et al., 2013; Tulpin et al., 2023; Tong et al., 2024).

*Feeling safe was associated with being seen and heard, recognised as an individual with individual needs, involvement on their own terms and respect and openness from the family and social and professional networks they are surrounded by (**BERGERSEN et al., 2022**).*

Time spent with peers who shared similar experiences, alongside support from family, fostered a sense of unity, as families often came together to navigate the emotional landscape collectively (ALMARZA and SMITH, 2010; Bergersen et al., 2022). In addition, friends were described as providing a vital sense of normalcy and a refuge from the stress of caregiving (Bergersen et al., 2022). Friendships allowed young adult caregivers to temporarily escape their emotional burden and receive both social and emotional support (Bergersen et al., 2022). Although friends could provide comfort, however, some young adult caregivers feared becoming a burden to them (Bergersen et al., 2022). Others described difficulties communicating with friends and finding interactions disappointing when friends failed to understand or engage, preventing them from sharing their thoughts and memories when support was most needed (Bergersen et al., 2022; Karlsson et al., 2013; McPhail et al., 2017). As one participant expressed:

*You realize who your true friends are because you see the people who keep in touch and check on you, but then there are people who don’t… You realize who’s going to be there for you when you need it (McPhail, 2027 P209).*

Some young adult caregivers expressed feeling frustrated and even provoked when friends discussed trivial problems (Bergersen et al., 2022).

Further, while the role of healthcare professionals was acknowledged, responses regarding their impact varied. Some young adult caregivers felt that healthcare professionals played a neutral or distant role in their caregiving journey, while others found support through more engaged or empathetic interactions (Karlsson et al., 2013; Tulpin et al., 2023; Tong et al., 2024).

Finally, young adult caregivers highlighted how a lack of support from employers and educational institutions could lead to feelings of hopelessness, prompting some to seek alternative employment to be present for their parents.

*Some struggled with moving away from home during their parents’ illness, while others struggled with motivation and chose to discontinue their studies (Bergersen, 2022, P. 6).*

*The silent struggle: Barriers to asking for support and sharing needs*

Despite the critical importance of support, many young adults reported barriers to expressing their needs or asking for support during their caregiving journey. Some young adult caregivers expressed a reluctance to share their emotions or ask for help, believing that doing so might impose on their friends, family, or peers (Fletcher et al., 2019; Bergersen et al., 2022). They also found it difficult to share their feelings, often due to a fear of burdening others or a desire to maintain control over the situation.

*I’m less worried about myself. I’m more worried about my mother! … I’m more concerned about whether her cancer has been controlled (**FANG et al., 2022**).*

Maintaining control required practical knowledge and support in terms of communication with both the ill parent and others around them (Karlsson et al., 2013). Some young adult caregivers experienced ‘misunderstood care’, in which both the parent and the young adult tried to protect each other by not sharing their emotions or concerns.

*Many also felt that their parents were trying to protect them and that they were not always completely honest (Bergersen, 2022, P. 6).*

*Information, communication, and involvement in navigating the disease trajectory and caregiving*

Young adult caregivers faced significant gaps in caregiving support, particularly in areas like knowledge and competence related to carrying out care tasks (Fang et al., 2022; ALMARZA and SMITH, 2010). Several young adult caregivers expressed feelings of being unprepared or incompetent in fulfilling caregiving responsibilities, especially related to administering medications, understanding medical procedures, or managing complex healthcare needs (Bergersen et al., 2022; Fang et al., 2022). Without proper guidance or training, they often felt overwhelmed, unsure, or incapable of providing the care required by their ill parent. This lack of knowledge and confidence in caregiving duties contributed to emotional strain, as young adult caregivers struggled with the responsibility of making important decisions or handling medical tasks with minimal support or direction (Fang et al., 2022; ALMARZA and SMITH, 2010).

Many young adult caregivers reported being excluded from key medical discussions or decisions, either because healthcare professionals did not involve them in the process or because family members took over the decision-making (Fang et al., 2022; Karlsson et al., 2013). This exclusion left them feeling disempowered and isolated, as they were prevented from actively contributing to their parents’ care. The lack of involvement intensified their sense of being left out and made them feel unsupported in navigating their new role as caregivers (Fang et al., 2022; Karlsson et al., 2013). Additionally, young adult caregivers who felt uninformed or excluded from caregiving decisions were more likely to struggle with feelings of helplessness and incompetence, which impeded their ability to cope (ALMARZA and SMITH, 2010; Kastrinos et al., 2023). In contrast, those who were better informed and involved in caregiving decisions were more able to seek support from others and manage their emotional responses to the situation (Kastrinos et al., 2023; Tulpin et al., 2023).

*In addition to EOL [end of life] information, young adult caregivers reported that parents’ sharing the details of their illness and treatment information mattered. Such disclosures allowed them to take on more caregiving responsibilities, which then helped them cope (Kastrinos, 2023, P. 418).*

Misunderstandings about caregiving and the importance of discussing the illness and plans with parents were also central to this theme (Karlsson et al., 2013). Some young adult caregivers reported feeling disconnected from their parents’ experience due to a lack of open conversation regarding the illness, future prognosis, and end-of-life decisions. This absence of dialogue could result in misunderstandings about the severity of the illness and the challenges ahead, leaving young adult caregivers feeling uncertain and unprepared for both the emotional and the practical aspects of caregiving (Bergersen et al., 2022; Fang et al., 2022; Kastrinos et al., 2023).

*I wish I had known the truth because at the end of the day, I [still] dropped my school. I left my job. … And I would have been spending more time with my dad (Kastrinos, 2023, P. 420).*

However, not all young adult caregivers wanted to be deeply involved in caregiving or to receive extensive information about their parents’ condition. Some preferred to distance themselves from the details, either as a coping mechanism or to maintain a sense of normalcy. For these young adult caregivers, too much information could feel overwhelming, adding to their stress rather than easing it (Kastrinos et al., 2023).

### 4.4 Confidence in summary findings

An overall assessment of confidence in each of the three themes was conducted using the GRADE CERQual approach. All studies had a high overall CERQual rating, with minor concerns regarding methodological limitations, relevance, coherence, and adequacy. Table 5 presents a summary of the qualitative findings and confidence assessments.

**Table 5:** Summary of qualitative findings and confidence assessment.

Review findings	Contributing studies	Confidence in the evidence	Explanation of confidence in the evidence assessment
The emotional burden of having a parent with cancer and the impact on the Young adult caregivers’ identity			
*The emotional burden of having a parent with cancer*	(26–36)	High	Ten studies with minor concerns regarding methodological limitations, relevance, coherence, and adequacy. High dependability
*Navigating care responsibilities, guilt, and boundaries*	(26–37)	High	Twelve studies with minor concerns regarding methodological limitations, relevance, coherence,and adequacy. High dependability
*Impact on identity and life priorities*	(26, 29, 31, 32, 34, 36, 37)	High	Seven studies with minor concerns regarding methodological limitations, relevance, coherence, and adequacy. High dependability
Balancing family dynamics: navigating conflicts, fostering closeness, and managing responsibilities			
*Family dynamic in transition*	(27, 28, 30, 32–34, 36, 37)	High	Eight studies with minor concerns regarding methodological limitations, relevance, coherence, and adequacy. High dependability
*The challenge of balancing caregiving and personal aspirations*	(26- 28, 31, 33, 35,37)	High	Seven studies with minor concerns regarding methodological limitations, relevance, coherence, and adequacy. High dependability
*Closeness & Conflicts*	(26, 28, 31–34, 37)	High	Sevenstudies with minor concerns regarding methodological limitations, relevance, coherence, and adequacy. High dependability
Effective management strategies: the role of support, information, and active involvement			
*The critical role of receiving support from family, friends, and healthcare professionals*	(26, 29, 32–34, 36)	High	Six studies with minor concerns regarding methodological limitations, relevance, coherence, and adequacy. High dependability
*The silent struggle: barriers to asking for support and sharing needs*	(28, 29, 31, 36),	High	Four studies with minor concerns regarding methodological limitations, relevance, coherence,and adequacy. High dependability
*Information, communication and involvement in navigating the disease trajectory and caregiving*	(26, 28,29, 32, 35, 36)	High	Six studies with minor concerns regarding methodological limitations, relevance, coherence, and adequacy. High dependability

## 5 Discussion

This systematic review, which comprised 12 studies, aimed to systematically identify and synthesise qualitative research on the lived experiences of young adults caring for a parent with cancer. The review explored two key questions: (1) how young adult caregivers experience the role of caring for a parent with cancer and (2) how young adult caregivers describe their everyday lives, including changes in roles, employment, education, and relationships.

In acknowledging the profound and challenging distress experienced by young adult caregivers, this review identified three key themes that illustrate how their daily lives change instantly upon a parent’s cancer diagnosis. Consistent with emerging literature, this shift leads to a multidimensional caregiver burden that affects individuals physically, emotionally, socially, and financially during a dynamic period of their lives (Waters et al., 2021). Impacts include shifting roles and relationships with other and balancing conflicting caregiving responsibilities with personal, educational, and professional commitments, often resulting in emotional distress, social isolation, and career disruption. On the other hand, caregiving can also have positive effects, such as personal growth. Factors that may contribute to these positive outcomes include female gender and religious beliefs: a finding also highlighted by Kang et al. (2013), who additionally identified advanced age and a spousal relationship as being associated with positive caregiving experiences (Kang et al., 2013).

The findings illustrate how communication, involvement, and the sharing of information play critical roles in enabling young adult caregivers to cope effectively with their caregiving responsibilities. Clear and transparent communication helps young adult caregivers feel supported, competent, and empowered in their role, whereas a lack of information or the withholding of key details may increase emotional strain and hinder their ability to manage their responsibilities. Effective communication with parents, healthcare professionals, and family members is essential for navigating the caregiving experience with a sense of agency and emotional resilience. Thus, our findings suggest that young adult caregivers may benefit from future research and supportive services that take a broader approach, focusing on larger and more comprehensive social support networks.

These needs are fundamentally similar to those of adult caregivers, who have expressed, for example, a strong desire for the timely provision of relevant information and a closer connection with healthcare professionals due to rapidly changing needs as the disease progresses (GULDAGER et al., 2022). Nevertheless, the impact of caregiving on young adults differs from that experienced by adult caregivers, particularly with respect to developmental stages and life expectations. The negative impacts of cancer caregiving may be amplified for young adult caregivers when caregiving responsibilities overlap and, at times, conflict with the key developmental tasks of young adulthood (Waters et al., 2021). Furthermore, previous research has found that young adult caregivers are more likely than adult caregivers to experience psychological distress and strain as a result of their caregiving role, as well as to perceive traumatic events as more stressful (Kang et al., 2013).

This review found no studies focusing specifically on cancer diagnoses with rapid progression and poor prognosis. Among the most aggressive forms of cancer is that of primary malignant brain tumors, which are characterised by a poor prognosis and a five-year survival rate of only 5% (Ostrom et al., 2021). Having a supportive family and functioning as a cohesive unit are crucial in such situations. The disease often leads to cognitive decline, personality changes, and physical disabilities, which require continuous caregiving. These challenges underscore the importance of supporting families as integrated systems and highlight the need for further research to identify effective interventions, tailored support, and guidance to help young adult caregivers navigate their roles more effectively. Future research should focus on more aggressive cancer types, as the severity and progression of the cancer diagnosis are likely to influence the level of caregiver burden.

### 5.1 Strengths and limitations

To our knowledge, this is the first systematic review to comprehensively identify and synthesise qualitative research on young adults’ lived experiences of parental cancer. The literature search was thorough and conducted in collaboration with an information specialist to ensure its quality. Apart from minor deviations, the review adhered to a prepublished protocol to maintain a rigorous and systematic approach. However, this review has some limitations. First, despite an extensive literature search, it is possible some relevant studies were missed. Nonetheless, our goal was to provide an overview of the evidence, and it is unlikely that the inclusion of a few additional studies would have significantly altered our overall findings. Second, a significant methodological limitation concerns age-related eligibility and reporting. Although young adulthood was defined as 18–35 years (Arnett, 2000), several included studies reported broader age ranges or incomplete age information. The decision to include studies in which the majority (>50%) of participants fell within the defined age range, or where qualitative data specific to this age group could be extracted separately, was a pragmatic response to limitations in the existing qualitative literature. However, this approach may reduce the specificity of the findings and limit their transferability to young adults as a distinct developmental group. Third, a significant challenge was the lack of data, as many studies did not report essential study characteristics. We deemed contacting the authors for additional information excessive and believed it unlikely that doing so would result in relevant high-quality data due to the studies’ poor reporting. Finally, the subjective nature of qualitative research means that the synthesis is influenced by the interpretations of the reviewers, which could introduce bias.

## 6 Implications for nursing practice

This study highlights the unique challenges faced by young adults experiencing parental cancer, emphasising their emotional burden, shifting identities, and caregiving responsibilities. Synthesising qualitative research provides valuable insights for healthcare professionals to develop targeted interventions that support young adults’ mental well-being, family dynamics, and management mechanisms. The findings highlight the need for enhanced psychosocial interventions. They may inform the development of policies and clinical practices that incorporate psychological support and structured guidance within oncology care, thereby improving the quality of life among young adult caregivers.

Healthcare professionals should proactively assess the emotional and psychological well-being of young adults with a parent diagnosed with cancer, integrating routine screening for anxiety, depression, and caregiver burden. Recognising the dual role of such young adults as both individuals and caregivers, clinicians should encourage open family communication and guide managing conflicts and shifting responsibilities. Additionally, empowering young adults with personalised strategies and self-care practices can help prevent burnout and protect their aspirations and well-being.

## 7 Conclusion

In conclusion, the findings indicate that young adults live everyday lives that unfold in parallel with the medical trajectory of their parent, a process characterised by shifting social roles, disrupted relationships, and an evolving sense of identity. In this context, young adult caregivers experience profound transformations in their identities as they navigate the dual pressures of caregiving and personal development. The sense of lost freedom and overwhelming responsibility often mark a critical juncture in their lives, leading to exhaustion and caregiver fatigue. The competing demands of education, work, relationships, and caregiving compel young adult caregivers to develop management strategies and reassess their priorities, ultimately reshaping their sense of self. The emotional burden, compounded by societal and familial expectations, creates a complex landscape in which young adult caregivers must reconcile hopes and fears. Although these challenges can be overwhelming, they may also catalyse personal growth, prompting a reevaluation of values and a deeper appreciation for life. Nevertheless, difficulties in seeking or accepting support underscore the importance of targeted interventions to safeguard young adult caregivers’ well-being and help preserve their individuality amidst caregiving responsibilities.

## Funding information

No funding was received.

## Declaration of generative AI and AI-assisted technologies in the manuscript preparation process

During the preparation of this work the authors used Microsoft Copilot in order to enhance readability and language quality. After using this tool, the authors)reviewed and edited the content as needed and takes full responsibility for the content of the published article.

## Implication for practice

Healthcare professionals should screen for young adults' emotional distress, support family communication, and promote management strategies to prevent burnout and support their well being.

## Reporting method

The ENTREQ reporting guideline was used to support the reporting of this systematic review.

## Patient or public contribution

There was no patient or public contribution.

## Protocol registration

The protocol is registered in the International Prospective Register of Systematic Reviews with register number CRD42023492854.

## CRediT authorship contribution statement

**Rikke Guldager:** Writing – review & editing, Writing – original draft, Visualization, Validation, Methodology, Formal analysis, Conceptualization. **Trine Andersen:** Writing – review & editing, Writing – original draft, Visualization, Validation, Methodology, Funding acquisition, Formal analysis, Conceptualization. **Helle Sørensen von Essen:** Writing – review & editing, Writing – original draft, Visualization, Validation, Methodology, Formal analysis, Conceptualization. **Mette Gothardt Lundh:** Writing – review & editing, Writing – original draft, Methodology, Conceptualization. **Karin Bundgaard:** Writing – review & editing, Writing – original draft, Visualization, Validation, Methodology, Conceptualization. **Tina Wang Vedelø:** Writing – review & editing, Writing – original draft, Visualization, Validation, Methodology, Investigation, Formal analysis, Conceptualization.

## Declaration of competing interest

The authors declare that they have no known competing financial interests or personal relationships that could have appeared to influence the work reported in this paper.

## Figures and Tables

**Table:** 

#	Search
1	adult/ or young adult/ or Adult Children/ or Adolescent/ or Caregivers/ or exp parent-child relations/
2	(adult* or young people* or young person* or adulthood or adolesc* or son or sons or daughter* or offspring or caregiver* or caregiving).ti,ab,kf.
3	1 or 2
4	(parent* or mother* or father*).ti,ab,kf.
5	parents/ or fathers/ or mothers/ or family relations/ or exp parent-child relations/ or parenting/
6	4 or 5
7	(cancer* or neoplasm* or tumor* or tumour*).ti,ab,kf.
8	exp Neoplasms/
9	7 or 8
10	3 and 6 and 9
11	(experience* or need or needs or support or caring or preference* or reflection* or perception* or challenge* or strain or belief* or attitude* or perspective* or responsibili* or caregiver burden or empathy or burnout*).ti,ab,kf.
12	(psychological adj3 (distress or stress or condition*)).ti,ab,kf.
13	"Health Services Needs and Demand"/ or Empathy/ or Patient Preference/ or stress, psychological/ or burnout, psychological/ or caregiver burden/ or psychological distress/
14	11 or 12 or 13
15	10 and 14

**Table:** 

#	Search
1	adult/ or young adult/ or adult child/ or adolescent/ or caregiver/ or adulthood/ or daughter/ or son/ or progeny/ or exp child parent relation/
2	(adult* or young people* or young person* or adulthood or adolesc* or son or sons or daughter* or offspring or caregiver* or caregiving).ti,ab,kf.
3	1 or 2
4	(parent* or mother* or father*).ti,ab,kf.
5	parent/ or mother/ or mother child relation/ or father/ or father child relation/ or exp family relation/
6	4 or 5
7	(cancer* or neoplasm* or tumour* or tumor*).ti,ab,kf.
8	exp neoplasm/
9	7 or 8
10	3 and 6 and 9
11	(experience* or need or needs or support or caring or preference* or reflection* or perception* or challenge* or strain or belief* or attitude* or perspective* or responsibili* or caregiver burden or empathy or burnout*).ti,ab,kf.
12	(psychological adj3 (distress or stress or condition*)).ti,ab,kf.
13	experience/ or supportive care need/ or caregiver support/ or exp social support/ or care behavior/ or parental behavior/ or patient preference/ or attitude/ or attitude to death/ or family attitude/ or patient attitude/ or empathy/ or exp physiological stress/ or burnout/ or caregiver burnout/ or caregiver burden/ or distress syndrome/
14	11 or 12 or 13
15	10 and 14

**Table:** 

#	Search
1	((((((Index Terms: (empathy))))) *OR* ((((Index Terms: (psychological stress))))) *OR* ((((Index Terms: (burnout))))) *OR* ((((Index Terms: (psychological distress))))) *OR* ((((Index Terms: (needs))))) *OR* ((((Index Terms: (support))))) *OR* ((((Index Terms: (strain))))) *OR* ((((Index Terms: (caregiver burden)))))) *OR* (((((((title: (psychological)))))) *OR* (((((abstract: (psychological)))))) *OR* (((((Keywords: (psychological)))))))
2	((((((title: (distress))))) *OR* ((((title: (stress))))) *OR* ((((title: (condition*)))))) *OR* (((((abstract: (distress))))) *OR* ((((abstract: (stress))))) *OR* ((((abstract: (condition*)))))) *OR* (((((Keywords: (distress))))) *OR* ((((Keywords: (stress))))) *OR* ((((Keywords: (condition*)))))))) *OR* (((((Keywords: (experience*)))) *OR* (((Keywords: (need)))) *OR* (((Keywords: (needs)))) *OR* (((Keywords: (support)))) *OR* (((Keywords: (caring)))) *OR* (((Keywords: (preference*)))) *OR* (((Keywords: (reflection*)))) *OR* (((Keywords: (perception*)))) *OR* (((Keywords: (challenge*)))) *OR* (((Keywords: (strain)))) *OR* (((Keywords: (belief*)))) *OR* (((Keywords: (attitude*)))) *OR* (((Keywords: (perspective*)))) *OR* (((Keywords: (responsibilit*)))) *OR* (((Keywords: (caregiver burden)))) *OR* (((Keywords: (empathy)))) *OR* (((Keywords: (burnout*))))) *OR* ((((abstract: (experience*)))) *OR* (((abstract: (need)))) *OR* (((abstract: (needs)))) *OR* (((abstract: (support)))) *OR* (((abstract: (caring)))) *OR* (((abstract: (preference*)))) *OR* (((abstract: (reflection*)))) *OR* (((abstract: (perception*)))) *OR* (((abstract: (challenge*)))) *OR* (((abstract: (strain)))) *OR* (((abstract: (belief*)))) *OR* (((abstract: (attitude*)))) *OR* (((abstract: (perspective*)))) *OR* (((abstract: (responsibilit*)))) *OR* (((abstract: (caregiver burden)))) *OR* (((abstract: (empathy)))) *OR* (((abstract: (burnout*))))) *OR* ((((title: (experience*)))) *OR* (((title: (need)))) *OR* (((title: (needs)))) *OR* (((title: (support)))) *OR* (((title: (caring)))) *OR* (((title: (preference*)))) *OR* (((title: (reflection*)))) *OR* (((title: (perception*)))) *OR* (((title: (challenge*)))) *OR* (((title: (strain)))) *OR* (((title: (belief*)))) *OR* (((title: (attitude*)))) *OR* (((title: (perspective*)))) *OR* (((title: (responsibilit*)))) *OR* (((title: (caregiver burden)))) *OR* (((title: (empathy)))) *OR* (((title: (burnout*)))))))
3	((((((((Index Terms: (parent child relations)))))) *OR* (((((Index Terms: (adult children)))))) *OR* (((((Index Terms: (adolescence)))))) *OR* (((((Index Terms: (adolescent)))))) *OR* (((((Index Terms: (caregivers)))))) *OR* (((((Index Terms: (adult offspring)))))) *OR* (((((Index Terms: (adult))))))) *OR* ((((((title: (adult*))))) *OR* ((((title: (young people*))))) *OR* ((((title: (young person*))))) *OR* ((((title: (adulthood))))) *OR* ((((title: (adolesc*))))) *OR* ((((title: (son))))) *OR* ((((title: (sons))))) *OR* ((((title: (daughter*))))) *OR* ((((title: (offspring))))) *OR* ((((title: (caregiver*))))) *OR* ((((title: (caregiving)))))) *OR* (((((abstract: (adult*))))) *OR* ((((abstract: (young people*))))) *OR* ((((abstract: (young person*))))) *OR* ((((abstract: (adulthood))))) *OR* ((((abstract: (adolesc*))))) *OR* ((((abstract: (son))))) *OR* ((((abstract: (sons))))) *OR* ((((abstract: (daughter*))))) *OR* ((((abstract: (offspring))))) *OR* ((((abstract: (caregiver*))))) *OR* ((((abstract: (caregiving)))))) *OR* (((((Keywords: (adult*))))) *OR* ((((Keywords: (young people*))))) *OR* ((((Keywords: (young person*))))) *OR* ((((Keywords: (adulthood))))) *OR* ((((Keywords: (adolesc*))))) *OR* ((((Keywords: (son))))) *OR* ((((Keywords: (sons))))) *OR* ((((Keywords: (daughter*))))) *OR* ((((Keywords: (offspring))))) *OR* ((((Keywords: (caregiver*))))) *OR* ((((Keywords: (caregiving))))))))
4	(((((((Keywords: (parent*))))) *OR* ((((Keywords: (mother*))))) *OR* ((((Keywords: (father*)))))) *OR* (((((abstract: (parent*))))) *OR* ((((abstract: (mother*))))) *OR* ((((abstract: (father*)))))) *OR* (((((title: (parent*))))) *OR* ((((title: (mother*))))) *OR* ((((title: (father*))))))) *OR* ((((((Index Terms: (parenting)))))) *OR* (((((Index Terms: (parent child relations)))))) *OR* (((((Index Terms: (family relations)))))) *OR* (((((Index Terms: (mothers)))))) *OR* (((((Index Terms: (fathers)))))) *OR* (((((Index Terms: (parents))))))))
5	(((((((title: (cancer*))))) *OR* ((((title: (neoplasm*))))) *OR* ((((title: (tumor*))))) *OR* ((((title: (tumour*)))))) *OR* (((((abstract: (cancer*))))) *OR* ((((abstract: (neoplasm*))))) *OR* ((((abstract: (tumor*))))) *OR* ((((abstract: (tumour*)))))) *OR* (((((Keywords: (cancer*))))) *OR* ((((Keywords: (neoplasm*))))) *OR* ((((Keywords: (tumor*))))) *OR* ((((Keywords: (tumour*))))))) *OR* ((((((Index Terms: (neoplasms)))))) *OR* (((((Index Terms: (cancer)))))))))
6	1 AND 2 AND 3 AND 4 AND 5

**Table:** 

#	Search
S1	(((MH "Adult+") OR (MH "Adult Children") OR (MH "Adolescence") OR (MH "Caregivers") OR (MH "Parent-Child Relations+")) OR (TI adult* OR TI young people* OR TI young person* OR TI adulthood OR TI adolesc* OR TI son OR TI sons OR TI daughter* OR TI offspring OR TI caregiver* OR TI caregiving OR AB adult* OR AB young people* OR AB young person* OR AB adulthood OR AB adolesc* OR AB son OR AB sons OR AB daughter* OR AB offspring OR AB caregiver* OR AB caregiving))
S2	((TI parent* OR TI mother* OR TI father* OR AB parent* OR AB mother* OR AB father*) OR ((MH "Parent-Child Relations+") OR (MH "Parenting") OR (MH "Family Relations+") OR (MH "Parents+")))
S3	((TI cancer* OR TI neoplasm* OR TI tumor* OR TI tumour* OR AB cancer* OR AB neoplasm* OR AB tumor* OR AB tumour*) OR (MH "Neoplasms+"))
S4	((TI experience* OR TI need OR TI needs OR TI support OR TI caring OR TI preference* OR TI reflection* OR TI perception* OR TI challenge* OR TI strain OR TI belief* OR TI attitude* OR TI perspective* OR TI responsibili* OR TI caregiver burden OR TI empathy* OR TI burnout* OR AB experience* OR AB need OR AB needs OR AB support OR AB caring OR AB preference* OR AB reflection* OR AB perception* OR AB challenge* OR AB strain OR AB belief* OR AB attitude* OR AB perspective* OR AB responsibili* OR AB caregiver burden OR AB empathy* OR AB burnout*) OR (TI (psychological N3 (distress OR stress OR condition*)) OR AB (psychological N3 (distress OR stress OR condition*))) OR ((MH "Health Services Needs and Demand") OR (MH "Empathy") OR (MH "Patient Preference") OR (MH "Stress, Psychological") OR (MH "Caregiver Burden") OR (MH "Psychological Distress") OR (MH "Support, Psychosocial+")))
S5	S1 AND S2 AND S3 AND S4

**Table:** 

Category	Information	Description
Stage I. “Contextual” details		
*General information*	Author	Full name of first author
	Title	Verbatim extraction
	Year	Year of publication
	Study location	Country of study site
	Aim/objective	Verbatim extraction
*Setting*	Context	Context of the study (hospital, home, etc.)
*Population*	Studied population	Characteristics
*Methodology*	Study design	E.g., qualitative study, quantitative study, mixed-methods study
	Recruitment	E.g., convenience sampling, purposive sampling
	Data collection method(s)	E.g., focus group interview, individual interviews
	Data analysis	E.g., grounded theory, content analysis
Stage II. Extraction on the “findings”		
*Results*	Findings	Verbatim extraction on e.g. quotes from participants, author interpretations, themes, and sub-themes

**Table:** 

KEY CHARACTERISTICS OF STUDY					STUDY POPULATION (YA)						Gender, female (%)	
Author, publication year	Country and continent of corresponding author	Aim/objective	Study design	Context	Total number of participants (YA)	Cancer diagnosis of parent	Age at diagnosis (mean)	Occupation	Housing situation	Primary caregiver	YA	Parent
Almarza (2008)	Canada, North America	Explore the lived experience of adult children living at home whose parent has been diagnosed with, and treated for, cancer	Qualitative study: semi-structured interviews	Home	3	Acute myelogenous leukemia, urinary	23,7	Student, employed	Living at home with parent	No	66,7	0
Bergersen et al. (2022)	Norway and Sweden, Europe	Explore adolescents’ and young people’s needs and preferences for support as they live with a parent with life-threatening cancer	Qualitative study, grounded theory approach with dialogic interviews	Home	10	Bowel-, ovarian-, cervical-, brain-, abdominal-, breast-, bone marrow- and neuroendocrine	NA^a^	Student, employed	Living at home with parent	No	70	70
Fang et al. (2022)	Taiwan Asia,	Understand daughters’ lived experiences after their mothers were diagnosed with breast cancer	Qualitative study, in-depth interviews	Abroad/ home	18	Breast	NR^b^	Student, employed	Living at home with parent, living away from parent	No	100	100
Fletcher et al. (2019)	Australia, Oceania	Explore young people’s perceptions of their parent’s cancer using the Common-Sense Model of Self-Regulation as a theoretical framework	Qualitative study, semi-structured interviews	Home	11	Non-Hodgkins lymphoma-, lung-, bowel-, pancreatic-, kidney-, testicular-, ovarian-, cervical- and breast	NA^a^	NR^b^	Living at home with parent	Yes	63,6	NR^b^
Fujimoto and Kanda (2023)	Japan, Asia	Reveal the experiences of adolescent daughters who are beginning to live with their mothers’ cancer	Qualitative study, semi-structured interviews	Home	14	Breast	NR^b^	Student	Living at home with parent	No	100	100
Goldblatt et al. (2019)	Israel, Middle East	Explore the experiences of young adults, who acted as caregivers for a dying parent	Qualitative study, demographic questionnaires, and semi-structured interviews	Home	14	Terminal, unknown type	NA^a^	NR^b^	Living at home with parent, living away from parent	Yes	64,3	NR^b^
Karlsson et al. (2013)	Sweden, Europe	Describe young adults’ own perspectives on the experience of having a parent with cancer	Qualitative study, narrative interviews	Home	6	Unknown type	15,3	NR^b^	Living at home with parent, living away from parent	Yes	83,3	NR^b^
Kastrinos et al. (2023)	USA, North America	Identify factors that play a role in how parents’ information sharing and withholding affects EYACs’ caregiving and coping experiences across the disease trajectory, including during bereavement	Qualitative study, semi-structured interviews. Part of a larger, convergent parallel mixed-method study	Online recruitment from grief and support groups	33	Adenocarcinoma-, bile duct-, brain-, breast-, esophageal-, gastric-, kidney-, lung-, lymphoma-, neuroendocrine-, pancreatic-, rectal- and skin	26,39	NR	NR	NR	57,58	36,36
McPhail et al. (2017)	Canada, North America	Explore emerging adult, university students’ (a) perceptions and beliefs about cancer, its causes, and prevention strategies, (b) perceived outcomes of their lived experience with parental cancer, (c) life as a university student when experiencing parental cancer, (d) coping strategies and support systems used during this experience, and (e) personal health beliefs and behaviors since experiencing parental cancer	Qualitative study, using semi-structured interviews	Private location, usually their home	17	Unknown type	Between 18–27	students	Living at home with parent, living away from parent	NR	82	NR
Puterman and Cadell (2008)	Canada, North America	Explore how young adult women experience a parent’s cancer diagnosis and treatment	Qualitative study, autoethnographic approach using interactive, conversational interviews	Publicly funded cancer treatment center	5	Breast-, lymphoma-and kidney	21,4	NR	NR	NR	100	66,67
Tong et al. (2024)	Canada, North America	Explored the experiences of EAs caring for parents living with advanced disease."	Qualitative study, grounded theory approach with semi-structured interviews	Online	12	Advanced cancer (50%) Severe arthritis, Alzheimer’s disease, Coronary heart disease/heart failure	26	Student, employed	Living at home with parent, living away from parent	Yes	100	58
TULPIN et al. (2023)	Belgium	To gain insight into the perceived support needs of AYAs who have a parent with cancer, including the mentioned underrepresented subgroups, and the perceived factors facilitating or hindering their use of support	Qualitative study, using semi-structured interviews	Home	17	Breast cancer, Leukemia, Lymphatic cancer, Colon cancer, Brain cancer, Bile duct cancer, Esophageal cancer	NR	NR	NR	No	65	47

^a^ NA: Not applicable.

^b^ NR: Not reported.

## Appendix I: Draft search strategy

MEDLINE (Ovid) 〈1946 to September 04, 2025〉

Embase 〈1974 to September 04, 2025〉

PsycINFO_search_080925

Cinahl (EBSCO) 〈1974 to September 04, 2025〉
